# The I. H. Association

**Published:** 1882-04

**Authors:** 


### HOMŒOPATHIC PHYSICIAN.

A MONTHLY JOURNAL OF MEDICAL SCIENCE.

"If our school ever gives up the strict inductive method of Hahnemann, we are lost, and deserve only to be mentioned as a caricature, in the history of medicine."—CONSTANTINE HERING.

Vol. II. APRIL, 1882. No. 4.

### EDITORIAL.

THE I. H. ASSOCIATION: It is gratifying to note the increasing interest manifested by homœopathists in the I. H. Association. This interest is shown by frequent letters of inquiry, seeking information concerning its objects, work, etc. As an answer to all such inquiries, we give a brief and simple statement of *our* view of the object and work of the Association.

Dr. Smythe, an allopath, tells us that: "Carroll Dunham's address was only the exciting cause of the schism which took place in the ranks of Homœopathy. It has been gathering form for a long time, and must have come sooner or later; in fact, it could not have been delayed much longer. There are now two wings to the school, the liberals and the straight-jackets. A house cannot stand which is divided against itself. The liberals will necessarily become eclectics, and the straight-jackets will return to Hahnemannism, pure and unadulterated. Preliminary steps to accomplish this end have already been taken. During the meeting of the American Institute, at Milwaukee, June, 1880, the International Hahnemannian Association was formed. * * * The formation of this Association, and the adoption of its platform is a return to the pure inflexible, dogmatic Homœopathy of Hahnemann."

The above quoted paragraph, though from the pen of an allopath, tells fairly well the reasons which led to the formation of the I. H. Association. To preserve and develop "the pure inflexible, dogmatic Homœopathy of Hahnemann." What the association considers to be "the Homœopathy of Hahnemann," we may learn from the following:

"Essential Tenets of the Homœopathic Healing-Art.—The cure of the sick is most easily, mildly and permanently effected by medicines that are themselves capable of producing in a healthy person morbid symptoms similar to those of the sick.

"'The only proper way to ascertain the sick-making properties of medicines is to prove them on the healthy.

'The changed and morbid conditions of tissues and organs are *results* of a dynamic disturbance, and not the *cause* of the disease.

'The totality of the symptoms, subjective and objective, is the sole indication for the choice of the remedy.

'In order to secure the best possible practical results, medicines must be administered singly, and in a dose just sufficient to cure.

'And *local treatment* by medicated topical applications, in *non-surgical* cases, is not only unnecessary, but is apt to change the location of the disease, and induce dangerous complications, and never *permanently* cures.'

"These principles guided Hahnemann, Hering, Dunham, and the early pioneers, and are still the best guiding principles and safest rules in practice."

However much we may regret that all cannot, or will not, see the truth as we see it, we must acknowledge the truth of Dr. Smythe's* assertion that there are two parties, with very different aims and purposes, each claiming the honorable title of homœopathists. Indeed, no one at all conversant with the current literature of the school, can doubt the existence of these factions. The one party tending to eclectic methods and practices; the other adhering to the principles and practice of Hahnemann. Then it is evident that the question which divides these parties is not one of "high" or "low" potency practice, as some would have it. It is deeper than that; it is more potent than that. It is a question of law versus empiricism.

The question at issue then being so plain, the decision as to the side one should assist ought also to be easy and plain. Let each

---

*We would only dissent from Dr. Smythe's description of the Homœopathy, we advocate, so far as to say that it is neither dogmatic nor inflexible. Homœopathy is based on a law of nature, therefore, it is not dogmatic and cannot be inflexible in its practical adaptation. Yet, had we to choose between a dogmatic assertion of Samuel Hahnemann, and that of any allopath or "liberal homœopath," we would most decidedly pin our faith to the *ipse dixit* of Hahnemann.

practitioner judge for himself, whether or not he is eclectic in principle, and desires to see eclecticism again rule in therapeutics; or, let him consider whether or not he believes in the law of the similars, and desires to see its use developed and perpetuated. As he conscientiously answers these questions, he decides whether or not he should be one of "the liberals," who, Dr. Smythe declares, "will necessarily become eclectics," or one of the "straight-jackets," who adhere to "Hahnemannism, pure and unadulterated." And let him also remember, that in a contest between truth and error, he who labors not for the truth, practically aids and abets the false. Says a writer: "Among our gravest sins of omission, we may count that of failing to fight for the truth when it is assailed." No true friend to Homœopathy can fail to see which party and practice he should join.

The International Hahnemannian Association was formed for the purpose of studying and developing pure Homœopathy in contradistinction to that which is mixed, partial or eclectic. Though there are many local societies, there is no *general* society in existence organized for this purpose. As many are seeking to engraft pathological and other hypotheses upon Homœopathy, it is evident that there should be a general organization designed to thwart any such mutilations, and to perfect Homœopathy after the pattern given us by Hahnemann. In forming this association for the defence and study of true Homœopathy, no assumption of superior wisdom, no arrogant challenge of defiance nor vain boast of censorship is made. While allowing to others the right to think and act as they please, we claim equal privileges for ourselves. We would not if we could, and certainly could not if we would, compel all to think and practice as we do. Yet, we do claim—and challenge others to refute the claim—that our system of practicing medicine is the most scientific in its principles, the quickest in its results, and the easiest in its methods, of any system that exists, or has ever been known. We go farther, and claim that our principles—the law of the similars, the single remedy and the minimum dose—*alone* represent Homœopathy. Yet, we by no means assert that this association numbers among its members all the true homœopathists; that none others are true homœopaths. There are many good and true men outside of its membership; but we do assert that these principles entirely and fully represent Homœopathy, as do no other.

We have said that we make no vain boast of censorship. For this reason: Those who practice according to the law and its corollaries, as just given, are true homœopathists, and with these we certainly can have no quarrel. Those who do not so practice *are not homœopathists*, no matter what may be their party name; with eclectics or allopathists, homœopathists can have nothing in common either in agreement or for dispute. A man's practice, not his party designation, makes him a homœopath, an allopath or eclectic.

There must ever be strife between truth and error, between law and empiricism; but this need not be personal. As a celebrated physician has said in another connection: "I trust I have made the issue perfectly distinct and intelligible. And let it be remembered that this is no subject to be smoothed over by nicely adjusted phrases of half-assent and half-censure, divided between the parties. The balance must be struck boldly and the result declared plainly. * * * Let it be remembered that *persons* are nothing in this matter; there is no quarrel here between *men*, but there is a deadly incompatibility and exterminating warfare between doctrines."

